# A meta-analysis of the prevalence of African animal trypanosomiasis in Nigeria from 1960 to 2017

**DOI:** 10.1186/s13071-018-2801-0

**Published:** 2018-05-02

**Authors:** Paul Olalekan Odeniran, Isaiah Oluwafemi Ademola

**Affiliations:** 1 Department of Veterinary Parasitology, Faculty of Veterinary Medicine, University of Ibadan, Ibadan, Nigeria; 2 Division of Infection and Pathway Medicine, Deanery of Biomedical Sciences, University of Edinburgh, Edinburgh, EH8 9JZ UK

**Keywords:** Prevalence, Trypanosomes, Livestock, Tsetse, Nigeria

## Abstract

**Background:**

African animal trypanosomiasis is an economically significant disease that affects the livestock industry in Nigeria. It is caused by several parasites of the genus *Trypanosoma*. National estimates of the disease prevalence in livestock and tsetse flies are lacking, therefore a systematic review and meta-analysis were performed to understand the trend of the disease prevalence over the years.

**Methods:**

Publications were screened in Web of Science, Ovid MEDLINE, Global Health, EMBASE and PubMed databases. Using four-stage (identification, screening, eligibility and inclusion) process in the PRIMSA checklist, only studies that met the inclusion criteria for AAT and tsetse infections were analysed. Point estimates prevalence and subgroup analyses based on diagnostic techniques in livestock were evaluated at 95% confidence interval (CI).

**Results:**

A total of 74 eligible studies published between 1960 and 2017 were selected for meta-analysis. This covers the six geopolitical zones, involving a total of 53,924 animals. The overall prevalence of AAT was 16.1% (95% CI: 12.3–20.3%). Based on diagnostic techniques, the prevalence of AAT in cattle was highest in PCR followed by serology and microscopy while the highest prevalence in pigs was observed with serology. Out of 12,552 tsetse flies examined from 14 eligible studies, an overall prevalence of 17.3% (95% CI: 4.5–36.0%) and subgroup prevalence of 49.7% (95% CI: 30.7–68.8%), 11.5% (95% CI: 6.1–18.5) and 4.5% (95% CI: 1.8–8.8%) in *G. morsitans*, *G. tachinoides* and *G. palpalis*, respectively, were observed using the random effects-model.

**Conclusions:**

The prevalence of trypanosomes in both vectors and animal hosts was high in Nigeria. Therefore, further research on risk factors, seasonal and transhumance effects, vectoral capacity and competence are warranted for an effective control of AAT in Nigeria.

**Electronic supplementary material:**

The online version of this article (10.1186/s13071-018-2801-0) contains supplementary material, which is available to authorized users.

## Background

African animal trypanosomiasis (AAT) is caused by extracellular protozoan parasites of the genus *Trypanosoma* and it severely affects the livestock industry in Nigeria causing significant losses which ranges from a decrease in milk production to death [1]. The wide distribution of the disease is attributed to the abundance of its biological and mechanical transmitting vectors which are tsetse flies and biting flies, respectively [2]. All warm-blooded animals including wildlife species have been implicated in the transmission cycle of the disease [3]. The mature infective form of the parasite, metacyclic trypomastigote, is found in the invertebrate host where several reproductive and developmental stages takes place [4]. Trypanosomes evade the immune system of the host because it possesses a variable surface antigen (VSG) which prevents them from lysing by complement alternative pathway [5, 6].

Tsetse flies cover an approximately 80% of the landmass in Nigeria [7], hence AAT continues to thrive, and losses incurred have not reduced [8]. The prevalence of trypanosome infections in the tsetse flies is often neglected probably due to the intensive labour required for its evaluation [9, 10]. Dissection of tsetse flies remains the most common technique for detecting trypanosomes. However, serological and molecular techniques were assumed to detect higher levels of infection and genetic diversity [10, 11]. Diagnosis of AAT in Nigeria has relied on microscopy for decades [12], although the technique is not very sensitive [13, 14]. Hence, low prevalence reporting at a time was due to diagnostic technique used. Only a few studies in recent years have been reported using serology and PCR [11, 14–17].

At present, there are no national AAT and tsetse infection prevalence rates in Nigeria but there are lots of regional disjointed AAT data sets that ought to be amalgamated to provide a national AAT and tsetse trypanosome infection rate. The effort of the Pan African Tsetse and Trypanosomiasis Eradication Campaign (PATTEC-Nigeria) seems not to be felt across the country [18], because there is no concise information on the trend of the disease in various livestock. To the authors’ knowledge, there is no study which has addressed the overall prevalence of *Trypanosoma* species infection in livestock and the risk associated with these infections in Nigeria. Therefore, this systematic review and meta-analysis was performed to determine the prevalence of *Trypanosoma* spp. in relation to diagnostic techniques used in livestock. The purpose was to provide data sets that are important in assessing the success of AAT control programmes over time, particularly with the increasing demand on improving food security in an ever-increasing human population.

## Methods

This study was conducted in accordance with the PRISMA guidelines (Preferred Reporting Items for Systematic Reviews and Meta-Analyses) which was used to ensure inclusion of all relevant information in the analysis [19] (see Additional file 1: Table S1).

### Search strategy

Publications were screened in Web of Science, Ovid MEDLINE, Global Health, EMBASE and PubMed using the University of Edinburgh Library database. The last search was done on the 7th September 2017. Search terms were done in English and included: trypanosomes, bovine, small ruminant, porcine, horses, camels, tsetse, *Glossina*, trypanosomiasis and Nigeria. Reference lists of relevant articles were visually scanned through to locate any omitted study.

### Inclusion criteria

**Fig. 1:**
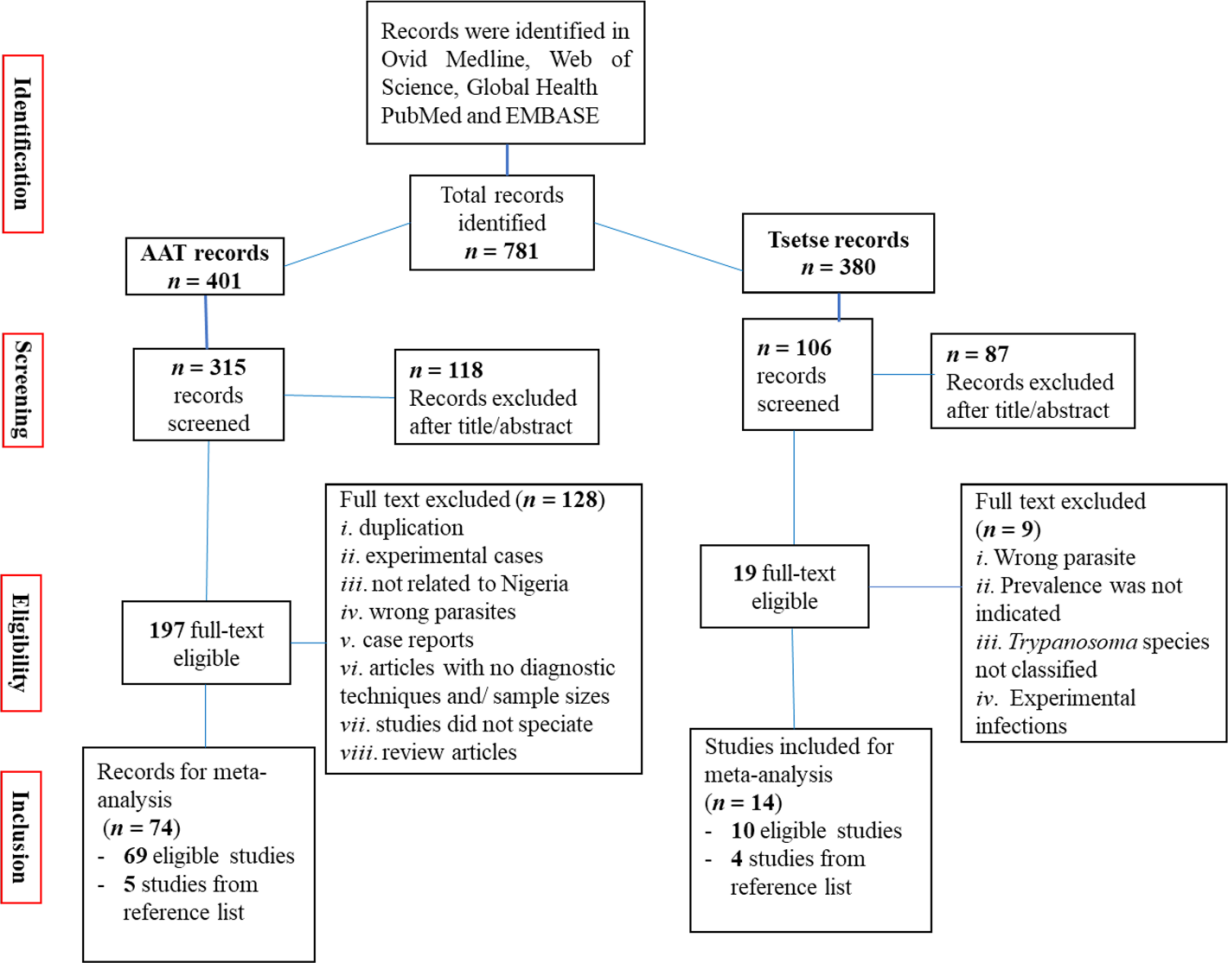
Flow diagram detailing the selection of eligible studies and excluded studies in a systematic approach for the prevalence of AAT and tsetse infections in Nigeria

A total of 781 published articles on AAT and tsetse infections were retrieved from the databases and reference lists of relevant studies assessed (Fig. 1). A total of 197 and 19 full texts on AAT and tsetse infections, respectively, were considered for eligibility screening (Fig. 1) after reading and sorting in Zotero Standalone (version 3.0.11). Inclusion criteria for meta-analysis were based on the following details: study type, location of study, tsetse species prevalence, *Trypanosoma* species prevalence, overall prevalence of trypanosomiasis in sampled herd, number of animal/tsetse examined, method of diagnosis and year of sampling. After assessing eligible studies, 74 and 14 studies representing AAT and tsetse infections, respectively, were included for meta-analysis. The total number of animals tested for AAT was 53,924 with a range of 55–7143 per study, while 12,552 tsetse flies were examined for trypanosomes. To evaluate the risk of bias, a quality assessment checklist was verified with some questions and given a score based on a scale of 0, 1 and 2 for no, yes and unclear, respectively.

### Statistical analysis

A summary of prevalence estimates was obtained using fixed and random effects models which were determined by the level of inconsistency/heterogeneity I^2^ statistic (inverse variance index). While a fixed model assumes perfect and equal procedures for all studies analysed, the random effects model explains the variation that could possibly occur among studies. The heterogeneity does not depend on chance nor the number of studies examined, with 25, 50 and 75% corresponding to low, moderate and high degree values of heterogeneity, respectively [20]. Heterogeneity was further investigated by arranging the studies based on relevant characteristics. Chi-square analysis was used to compare diagnostic methods used in the studies. Geographical locations (North/South) was a major subgroup analysis considered for both AAT and tsetse infections. Tukey’s *post-hoc* multiple pairwise comparison test of one-way ANOVA was used to compare study years of AAT prevalence reports. The map that shows AAT intensity was developed with qGIS (version 2.8.10). Microsoft Excel was used to manage raw data and calculate the 95% confidence intervals for descriptive analyses. Meta-analysis was conducted on both trypanosome infected animals and tsetse flies. All analyses were done using MedCalc® statistical software and WINPEPI statistic package (UK).

## Results

### Meta-analysis of AAT natural infection field-based studies

**Fig. 2:**
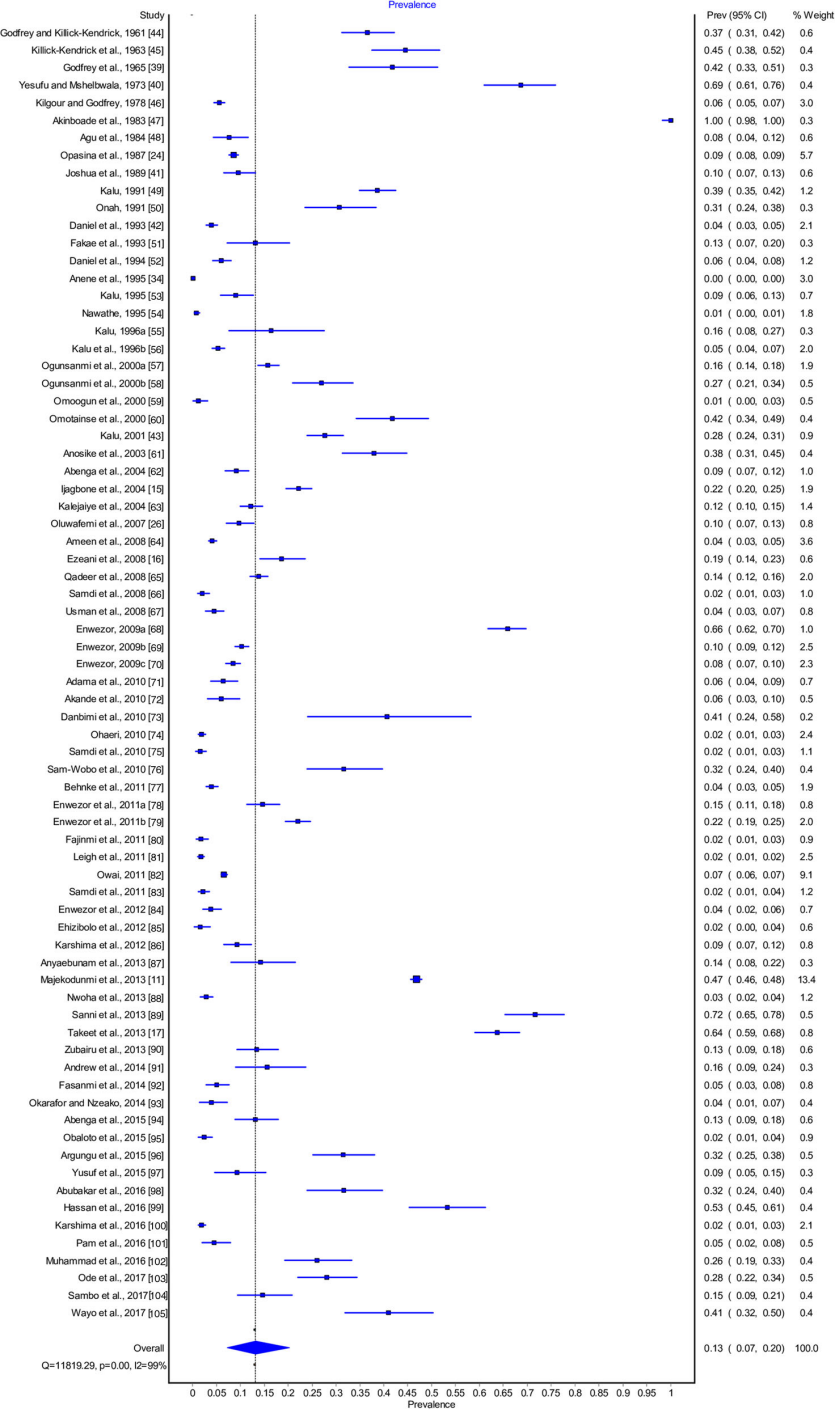
Forest plot of the prevalence estimates of AAT in animals in Nigeria between 1960–2017 [11, 15–17, 24, 26, 34, 39–105]

**Fig. 3:**
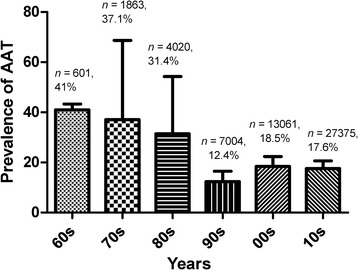
The prevalence of AAT in Nigeria over six decades. Tukey multiple pairwise comparison test of the analysis of variance shows no significant difference (*F*_(5, 68)_ = 1.616, *P* = 0.1676, *r*^2^ = 10.6%) in the prevalence reports across decades. Proportions of each study conducted (74 studies) and total number of animal screened is shown in the graph

The overall prevalence of AAT in the field studies (*n* = 74) was 16.1% (95% CI: 12.3–20.3%) (Table 1). Point estimates of individual studies are presented graphically in Fig. 2. A significant difference between study heterogeneity was observed (*χ*^2^ = 11830.2, I^2^ = 99.4, 95% CI: 99.3–99.4, *P <* 0.001). Sub-group analysis based on factors such as geographical region, type of animal, diagnostic technique and *Trypanosoma* species involved were further analyzed. Results are presented in Table 1. The prevalence of trypanosomes decreased in the first two decades after control intervention and increased thereafter with no significant difference in five decades (Fig. 3). AAT prevalence differed between regions with higher infection in southern Nigeria (*n* = 24; *χ*^2^ = 2890.4, I^2^ = 99.1, 95% CI: 99.1–99.3, *P* < 0.0001), though more studies have been reported in northern Nigeria (*n* = 54; *χ*^2^ = 8311.1, I^2^ = 99.4, 95% CI: 99.3–99.4, *P* < 0.0001). Cattle were the most studied animals (*n* = 55) with an overall prevalence of 17.0% (95% CI: 12.3–22.2%, *χ*^2^ = 9383.3, *df* = 54, I^2^ = 99.4, *P* < 0.0001), while the prevalence based on diagnostic technique revealed 13.0%, 21.0 and 25.5% for microscopy, serology and PCR methods, respectively. The analyzed results showed varying *Trypanosoma* species based on diagnostic techniques used for bovine trypanosomiasis. Microscopy showed higher prevalence *T. vivax*, while serology and PCR revealed a higher *T. congolense* (Table 1). The analysis of goat trypanosomiasis revealed more studies were carried out using microscopy. Caprine trypanosomiasis prevalence was 3.7% (*n* = 14) and 71.7% (*n* = 1) for microscopy and PCR, respectively. All studies on ovine trypanosomiasis were conducted using microscopy and demonstrated a prevalence of 7.7% (95% CI: 3.3–13.7%, *χ*^2^ = 453.1, *df* = 12, I^2^ = 97.4%, *P* < 0.0001) with *T. vivax* as most prevalent compared to the other species (Table 1). The prevalence of porcine trypanosomiasis observed was 3.2% (95% CI: 1.5–6.8%), 27.0% (95% CI: 21.2–33.7%) and 16.6% (95% CI: 14.0–19.5%) using microscopy, serology and PCR techniques, respectively. Reports on horses and camels are scarce, hence only one study each was retrieved and examined with prevalence of 1.7% (95% CI: 0.6–4.2%) and 31.5% (95% CI: 25.5–38.2%), respectively. Analyses of data in relation to decadal disease prevalence was used to understand the trend of AAT in Nigeria is shown graphically (Fig. 3).

**Table 1:** Amalgamated national (Nigeria) AAT prevalence 1960–2017

Attribute	No. of studies examined/total samples examined	Prevalence (95% CI) (%)	Measure of heterogeneity (Cochran’s Q)	% Variation; I^2^ (95% CI)	*P*-value
National	74 (53,924)	16.1 (12.3–20.3)	11830.19	99.4 (99.3–99.4)	*P* < 0.0001
Northern region	54 (32,134)	15.9 (11.1–21.4)	8311.08	99.4 (99.3–99.4)	*P* < 0.0001
Southern region	24 (22,055)	19.9 (14.0–26.5)	2890.36	99.2 (99.1–99.3)	*P* < 0.0001
Cattle	55 (40,863)	17.0 (12.3–22.2)	9383.29	99.4 (99.4–99.5)	*P* < 0.0001
Microscopy	45 (31,135)	13.0 (10.0–16.2)	2749.86	98.4 (98.2–98.6)	*P* < 0.0001
*T. vivax*	32 (16,942)	8.2 (5.7–11.1)	1183.98	97.4 (96.9–97.8)	*P* < 0.0001
*T. congolense*	32 (16,942)	2.8 (1.7–4.3)	725.89	95.7 (94.8–96.5)	*P* < 0.0001
*T. brucei*	32 (16,942)	1.7 (0.7–3.1)	1076.37	97.1 (96.6–97.6)	*P* < 0.0001
Serology	2 (1175)	21.0 (17.9–24.4)	1.57	36.4 (0.0–0.0)	*P* < 0.0001
*T. vivax*	2 (1175)	7.1 (5.7–8.7)	0.20	0.0 (0.0–0.0)	*P* = 0.67
*T. congolense*	2 (1175)	9.0 (7.4–10.7)	0.34	0.0 (0.0–0.0)	*P* = 0.60
*T. brucei*	2 (1175)	2.0 (0.7–4.1)	3.23	69.0 (0.0–93)	*P* = 0.07
PCR	7 (8672)	25.5 (10.5–44.4)	793.26	99.2 (99.0–99.4)	*P* < 0.0001
*T. vivax*	5 (8022)	9.5 (2.1–21.2)	231.15	98.3 (97.4–98.9)	*P* < 0.0001
*T. congolense*	3 (7754)	25.1 (16.7–34.4)	37.26	94.6 (87.9–97.7)	*P* < 0.0001
*T. brucei*	6 (8522)	4.5 (2.3–7.4)	56.50	91.2 (83.5–95.3)	*P* < 0.0001
Goats	15 (6270)				
Microscopy	14 (6065)	3.7 (2.2–5.5)	143.92	91.0 (86.6–93.9)	*P* < 0.0001
*T. vivax*	12 (5783)	1.3 (0.6–2.2)	84.21	86.9 (78.9–91.9)	*P* < 0.0001
*T. congolense*	12 (5783)	0.74 (0.3–1.3)	50.70	78.3 (62.6–87.4)	*P* < 0.0001
*T. brucei*	12 (5783)	0.59 (0.1–1.4)	94.37	88.3 (81.5–92.6)	*P* < 0.0001
PCR	1 (205)	71.7 (65.2–77.4)	–	–	–
*T. vivax*	1 (205)	71.7 (65.2–77.4)	–	–	–
Sheep	13 (4089)	7.7 (3.3–13.7)	453.06	97.4 (96.5–98.0)	*P* < 0.0001
Microscopy	13 (4089)	7.7 (3.3–13.7)	453.06	97.4 (96.5–98.0)	*P* < 0.0001
*T. vivax*	8 (2699)	2.6 (0.3–7.0)	198.47	96.5 (94.7–97.6)	*P* < 0.0001
*T. congolense*	8 (2699)	2.4 (0.7–5.1)	92.80	92.5 (87.5–95.5)	*P* < 0.0001
*T. brucei*	8 (2699)	1.1 (0.2–2.9)	74.50	90.6 (83.9–94.5)	*P* < 0.0001
Pig	2 (900)				
Microscopy	1 (189)	3.2 (1.5–6.8)	–	–	–
*T. brucei*	1 (189)	3.2 (1.5–6.8)	–	–	–
Serology	1 (189)	27.0 (21.2–33.7)	–	–	–
*T. brucei*	1 (189)	27.0 (21.2–33.7)	–	–	–
PCR	1 (712)	16.6 (14.0–19.5)	–	–	–
*T. congolense*	1 (712)	4.6 (3.3–6.4)	–	–	–
*T. brucei*	1 (712)	8.9 (7.0–11.2)	–	–	–
Horse	1 (243)	1.7 (0.6–4.2)	–	–	–
Camel	1 (200)	31.5 (25.5–38.2)	–	–	–

*Abbreviations*: *CI* confidence interval, *I* inconsistency or variation

### Meta-analysis of tsetse trypanosome infection field-based studies

**Fig. 4:**
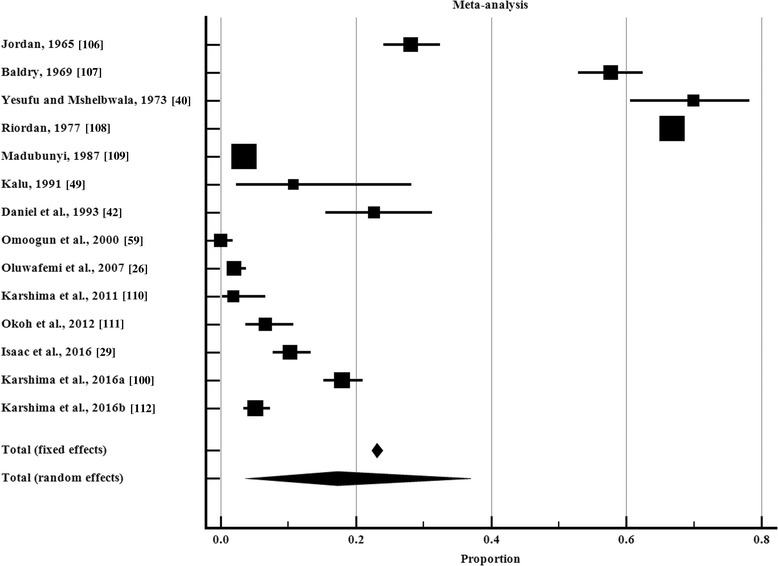
Forest plot of the prevalence estimates of trypanosome-infected tsetse flies in Nigeria between 1960–2017 [26, 29, 40, 42, 49, 59, 100, 106–112]

The overall prevalence of trypanosomes in tsetse flies captured in the field (*n* = 14) was 17.3% (95% CI: 4.5–36.0%) (Table 2). Point estimates of individual studies are presented graphically in Fig. 4. Significant prevalence heterogeneity was observed in the studies (*χ*^2^ = 6287.8, *df* = 13, I^2^ = 99.8, 95% CI: 99.8–99.9, *P* < 0.0001). The prevalence of trypanosomes in tsetse is significantly higher in the southern compared to northern Nigeria. There were significant (*F*_(2, 18)_ = 10.4, *P* = 0.01) differences in the levels of trypanosome infection between tsetse species. The prevalence of trypanosomes was highest in *G. morsitans* followed by *G. tachinoides* and *G. palpalis* (Table 2). Dissection under the microscope was the most frequent diagnostic technique with only few studies using PCR (Table 2). *Trypanosoma vivax* was mostly detected, followed by *T. congolense* and *T. brucei* in the tsetse species reported. No heterogeneity of prevalence was observed in *T. brucei* of *Glossina morsitans* (*χ*^2^ = 6.6, *df* = 4, I^2^ = 39.4%, 95% CI: 0.0–77.6%, *P* = 0.1586).

**Table 2:** Amalgamated national (Nigeria) tsetse trypanosome infection rates 1960–2017

Attribute	Number of studies examined/total samples examined	Prevalence (95% CI) (%)	Measure of heterogeneity (Cochran’s Q)	% Variation; I^2^ (95% CI)	*P*-value
National	14 (12,552)	17.3 (4.5–36.0)	6287.77	99.8 (99.8–99.9)	*P <* 0.0001
Northern region	9 (3107)	10.5 (5.1–17.5)	244.70	96.7 (95.3–97.7)	*P <* 0.0001
Southern region	5 (9445)	30.4 (0.6–78.6)	487.95	99.9 (99.9–100)	*P <* 0.0001
*G. morsitans*	5 (4883)	49.7 (30.7–68.8)	310.55	98.7 (98.1–99.1)	*P <* 0.0001
*T. vivax*	5 (4883)	36.8 (21.9–53.2)	221.35	98.2 (97.2–98.8)	*P <* 0.0001
*T. congolense*	5 (4883)	5.9 (2.6–10.3)	52.02	92.3 (85.0–96.1)	*P <* 0.0001
*T. brucei*	5 (4883)	0.2 (0.0–5.2)	6.60	39.4 (0.0–77.6)	*P =* 0.1586
*G. tachinoides*	11 (5793)	11.5 (6.1–18.5)	226.36	95.1 (93.1–96.6)	*P* < 0.0001
*T. vivax*	10 (5646)	4.9 (1.0–11.4)	263.30	96.6 (95.1–97.6)	*P* < 0.0001
*T. congolense*	10 (5646)	2.0 (0.5–4.5)	85.57	89.5 (82.8–93.6)	*P* < 0.0001
*T. brucei*	10 (5646)	1.2 (0.2–3.2)	77.30	88.4 (80.7–93.0)	*P* < 0.0001
*G. palpalis*	11 (1874)	4.5 (1.6–8.8)	118.42	91.6 (86.9–94.6)	*P* < 0.0001
*T. vivax*	6 (1610)	1.6 (0.3–3.9)	38.80	87.1 (74.3–93.6)	*P* < 0.0001
*T. congolense*	5 (1546)	1.5 (0.0–5.2)	69.91	94.3 (89.5–96.9)	*P* < 0.0001
*T. brucei*	5 (1546)	1.2 (0.0–5.2)	87.37	95.4 (91.9–97.4)	*P* < 0.0001

*Abbreviations*: *CI* confidence interval, *I* inconsistency or variation

## Discussion

### National AAT prevalence over the period 1960–2017

**Fig. 5:**
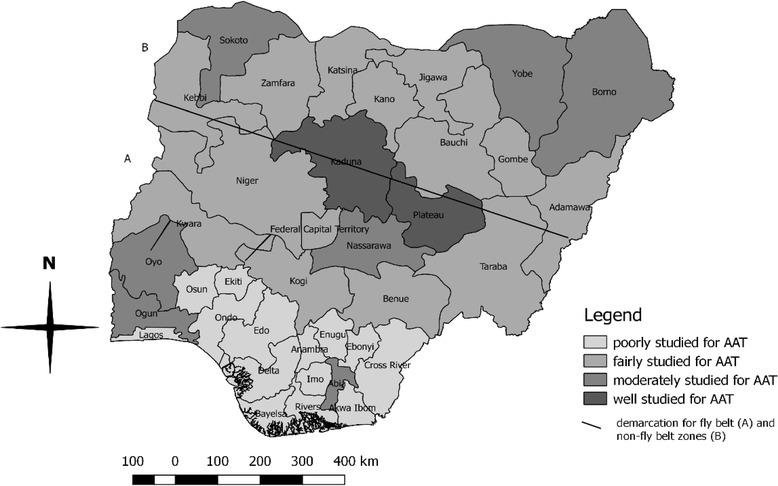
Nigeria states showing the intensity of trypanosomiasis studies conducted between 1960–2017

African animal trypanosomiasis (AAT) is a major threat to the livestock industry in Nigeria. The national prevalence of the disease is not known. The country has been involved in different elimination programmes of the disease since its first outbreak [21], and the PATTEC-initiative was also launched in 2001 [22]. To our knowledge, this is the first national report on the overall prevalence of AAT and trypanosome infected-tsetse flies after Nigeria gained independence in 1960. In order to understand the epidemiology of AAT, all studies on AAT were analyzed. The high prevalence of AAT observed in this study indicates that the disease is far from been eliminated. While it has been estimated that the presence of AAT reduces the total number of livestock in an area by 25–50%, it has also been predicted that with an elasticity of 0.2, AAT can reduce the agricultural gross domestic product (GDP) by 5–10% [18]. This study suggests an increasing trend of AAT in Nigeria and incidentally, well studied and moderately studied states for AAT have veterinary schools (Fig. 5).

### Regional (southern *vs* northern) AAT prevalence over the period 1960–2017

The prevalence of AAT observed in both regions showed no significant difference (Table 1), even though more studies were carried out in northern compared to the southern Nigeria with slightly higher prevalence of pooled estimates (Table 1). Most of the cases observed in the northern regions could be due to a substantial number of cattle herds in the region and transhumance activities [11]. Cattle in southern Nigeria are exposed to an abundance of tsetse flies; however, there is a significant amount of pasture and continuous treatment using chemotherapy. The prevalence in northern and southern regions showed that approximately all regions are at risk of infection (Table 1). Studies showed general reduction in AAT prevalence after control programmes were implemented in northern Nigeria [23] with a corresponding effect in southern Nigeria [24, 25]. However, due to lack of adequate surveillance, monitoring and control of the disease, the prevalence began to rise in northern Nigeria that was thought to be free of the vector [26]. Continuous movements and settlement patterns could be responsible for the increase in the prevalence of AAT in both regions.

### National tsetse-trypanosome infection rates over the period 1960–2017

Tsetse flies are known to be widely distributed across Nigeria [18]. About 196,000 km^2^ of landmass has been cleared in the northeastern region since 1967 [27] and an additional 1500 km^2^ in the north-central region in 1987 [28]. However, the presence of trypanosomes found in tsetse flies sampled from this cleared region in 2007 is significant in assessing the re-emergence of the infected flies [26]. A substantial heterogeneity existed among studies in northern and southern Nigeria (Table 2), even though the southern region is regarded as the “fly belt” zone, because of the favorable environmental condition for tsetse species. No study has compared tsetse infection in both regions as was done for AAT. It was observed that *G. tachinoides* have been mostly examined followed by *G. morsitans* and *G. palpalis*. The highest infection rate was observed in *G. morsitans* even though most of the flies were sampled before the new millennium. The collection of tsetse flies from year 2000 shows that the *Palpalis* group was mostly present compared to the *Morsitans* group. This could be due to human activities. The low prevalence trypanosomes observed in *G. palpalis* and *G. tachinoides* could be associated to the diagnostic technique (microscopy) used for detecting infection. Recently, PCR analysis has been used to detect trypanosomes in tsetse flies with a high presence of trypanosome DNA [29].

### Sub-group (goats, sheep, cattle and pigs) AAT prevalence over the period 1960–2017

Bovine trypanosomiasis has often been targeted because of the increased number of nomads and corresponding number of cattle in the country to meet the protein demand. Studies undertaken on cattle based on diagnostic technique, showed varied results for microscopy, serology and PCR methods (Table 1). This could have affected the disease perception at some point. Small ruminants such as sheep and goats have often been neglected in the integrated control approach because effort on trypanosome control has been concentrated on cattle. However, from this analytical study, the pooled prevalence suggests they could be of great interest in maintaining AAT in mixed herds (large and small ruminants). The low prevalence in small ruminants compared to cattle could be because most breeds of small ruminants in Nigeria such as Sahel/Desert/West African long-legged, West African dwarf and Sokoto goats [30] and Uda, West African dwarf, Balami and Yankasa sheep [31], are trypanotolerant. However, with the presence of trypanosomes in some of these breeds, it is necessary to consider the role of trypanotolerant small ruminants in the epidemiology of AAT. Porcine trypanosomiasis is also economically important especially with the recent detection of *T. brucei gambiense* in pigs from other countries [32, 33]. In Nigeria however, species observed were *T. b. brucei*, and *T. congolense* (Forest and Savannah). There is a dearth of information in the role of infected pigs in the epidemiology of the AAT in Nigeria. More studies are needed to determine possible transmission of trypanosomes from pigs to other animals and humans in Nigeria. The prevalence of trypanosomiasis in equines and camels suggest that more studies are needed to establish its overall prevalence.

### AAT prevalence heterogeneity over the period 1960–2017

The high heterogeneity index observed in livestock (Table 1) is suggestive of potential variations, which could be due to the characteristics of the type of animal sampled (e.g. sedentary, abattoir, market or institutional livestock), geographical regions, seasons of survey or diagnostic techniques used. From the meta-analysis, differences in prevalence exist in animals examined according to year and location of study. Most of the evaluated data from original studies did not investigate some variables such as status of animal while sampling, type of breed, tsetse-host contact, type of management practice and intervention factors, which could be used to identify the actual risk factors responsible for the infection in those animals. However, the prevalence of trypanosomes in tsetse flies which ensures continuous transmission could explain the increasing infection in animals. This persistence could even be sustained by mechanical vectors in the absence or presence of few tsetse flies [9, 34]. Hence, the prevalence variability is expected from the reports.

### AAT prevalence variability over the period 1960–2017: diagnostic techniques and prevalence estimates as a measure of variability and heterogeneity

Analysis shows that prevalence of AAT reduced from 1960s to 1990s (Fig. 3), this was due to a concerted government effort to oust tsetse flies the biological transmitting vector. Intense aerial spraying of DDT and dieldrin in northeastern Nigeria was reported to have occurred in 1967 because of the high incidence of the disease [28]. Between 1979 and 1987, there was BICOT project [26], which intensified elimination of tsetse flies in Lafia, Nasarawa State, hence a low prevalence was observed in 1990s (Fig. 3). However, an increase in AAT prevalence in the 2000s and 2010s, (Fig. 3), suggests the persistence of the disease despite the government’s efforts in the past.

Varying AAT prevalence was observed due to differences in methods of detection and identification of trypanosome species in Nigeria. Commonly observed methods were light microscopy, enzyme linked immunosorbent assay (ELISA) and polymerase chain reaction (PCR). Heterogeneity based on diagnostic technique showed that PCR was the most sensitive compared to serology and microscopy in all animals except pigs where serology was most sensitive. This could be due to the limited number of studies on porcine trypanosomiasis in Nigeria. The analysis in this study suggests that PCR is more sensitive than the other methods for AAT detection as observed in previous studies [14, 35].

There has been a great deal of debate on the prevailing *Trypanosoma* species. It was observed from this study that *Trypanosoma vivax* was mostly reported for studies examined with microscopy while those studied using serology and PCR methods revealed higher prevalence for *T. congolense*. It could be that *T. vivax* was seemingly recognizable or confused with other species under the microscope. The availability of internal transcribed spacer (ITS 1) primers that detects nuclear sequence gene makes identification of a wide range of *Trypanosoma* species possible [36]. *Trypanosoma brucei* maintained the lowest infection in cattle using the three techniques (Table 1). The commonly used primers for *T. brucei* in animals is TBR [37], while there has been argument on its sensitivity when compared to ITS1, and the later does not separate the *Trypanozoon* group which gives results for *T. brucei*, *T. evansi* and *T. equiperdum*.

Trypanosome prevalence in animals could be affected by several factors such as availability of reservoir hosts, seasonal factors, altitude, fly density and behavior, sensitivity of diagnostic technique, stage of infection, method of sampling, conflict and other human activities [11, 17, 38]. For instance, the microscopy techniques used by different authors varies, while wet mount, thin and thick smear was well-appreciated in the 1960s and 1970s [39, 40], more sensitive methods such as buffy coat technique, haematocrit centrifugation technique, sub-inoculation and standard trypanosome detection method were used in the 1980s, 1990s and 2000s onwards [41, 42, 43]. There have been arguments on the prevalence of *T. vivax* in dry and early wet seasons in Nigeria, while *T. congolense* have been detected more in the wet season [11]. Several reports in Nigeria have failed to specify the season of sampling in their methodology and this has made it difficult to classify seasonal reports as a measure of variability.

## Conclusions

The high prevalence of AAT and tsetse infection indicates that Nigeria may not eliminate trypanosomiasis any time soon if deliberate efforts are not employed. Microscopy has been widely used to investigate AAT and tsetse infection prevalence in Nigeria; however, the use of PCR could give a higher prevalence due to its sensitivity. Study methodology and risk factor assessment is necessary to validate research output. Further investigation is warranted on how this variation can be explained through risk factor assessment for AAT and tsetse infection.

## Additional file

Additional file 1: **Table S1.** PRISMA checklist. (DOCX 13 kb)
