# Supplementary material for: A meta-analysis of the prevalence of African animal trypanosomiasis in Nigeria from 1960 to 2017
Source: Parasit Vectors. 2018 May 2;11:280. doi: 10.1186/s13071-018-2801-0 (PMC5930763; doi:10.1186/s13071-018-2801-0)
Supplement: Supplementary file 1 — Table S1. PRISMA checklist. (DOCX 13 kb) [file 13071_2018_2801_MOESM1_ESM.docx]

**Prisma checklist**

Topic: It is described as meta-analysis

| Abstract | This is well-structured and it includes: background; objectives; data sources; study eligibility criteria, participants, and interventions; study appraisal and synthesis methods; results; limitations; conclusions and implications of key findings. |
| --- | --- |
| Introduction | The rationale for the review in the context of what is already known has been clearly stated, and an explicit statement of questions being addressed with reference to participants (livestock), interventions, comparisons, outcomes, and study design. |
| Methods | Eligibility criteria includes study characteristics and report characteristics (e.g., years considered, language, publication status) used as criteria for eligibility, giving rationale. All the information sources have been described (e.g., databases with dates of coverage, contact with study authors to identify additional studies) in the search and date last searched. Electronic search strategies of all database have been provided in supplementary file one including the limits used (1960–2017), such that it could be repeated. A figure has been presented inform of a flow chart which illustrates the selection criteria (screening, eligibility, inclusion). Data extraction process, assessment of bias, summary measures and measure of consistency were all included. All additional method of analysis (sub-group) have been clearly stated. |
| Results | Numbers of study screened, assessed for eligibility and included in the review was clearly stated. For each study, characteristics for which data were extracted (e.g., study size) have been clearly stated with necessary citations. Results of individual study have been presented. complete meta-analysis result was presented with all additional analysis. |
| Discussion | Findings have been properly summarised with strength of evidence for main outcome. The relevance of the report was also discussed with limitations. |
| Conclusions | General interpretation of the results in the context of other evidence, and implications for future research concludes the study report. |
| Funding | Funding bodies were properly acknowledged. |
